# Effects of Advanced Platelet Rich Fibrin (A-PRF+), Enamel Matrix Derivative (EMD) and Open Flap Debridement on clinical and wound healing parameters in molar furcation sites: A case series from a RCT study

**DOI:** 10.3389/fdmed.2023.1223217

**Published:** 2023-07-31

**Authors:** L. Pitzurra, D. Vasdravellis, N.A.M. Rosema, S. Bizzarro, B.G. Loos

**Affiliations:** Department of Periodontology, Academic Centre for Dentistry Amsterdam (ACTA), University of Amsterdam and Vrije Universiteit Amsterdam, Amsterdam, Netherlands

**Keywords:** periodontal reconstruction, PRF (platelet-rich fibrin), amelogenin (Emdogain), furcation defect, periodontal surgery

## Abstract

**Aim:**

To study the effects of advanced platelet-rich fibrin (A-PRF+) and enamel matrix derivative (EMD) compared to open flap debridement (OFD) alone in molar furcation sites grade II on clinical and wound healing parameters.

**Materials and methods:**

A randomized controlled trial was designed. Eligible patients were randomly allocated to one of three treatment groups: A-PRF+, EMD or OFD. The patients and clinical examiners were blinded for the treatment received. A minimally invasive microsurgical approach was performed for the three modalities. Clinical measurements were scored at baseline and 6 months post-operatively. The clinical healing of each furcation was scored via the Early Wound Healing Index on day 3, 1 week, 2 weeks and 6 weeks.

**Results:**

17 patients (A-PRF+ *n* = 6, EMD *n* = 5, OFD *n* = 6) completed the 6 months of follow-up. The further completion of the trial had to be cancelled due to the COVID-19 pandemic. In three patients in the A-PRF+ group, the grade II of the treated furcation regressed to grade I; the corresponding number in the EMD and OFD groups was zero and one respectively. Further, 3, 1 and 4 patients in the PRF, EMD and OFD groups respectively, showed a gain of bone level ≥1 mm. The defects in the A-PRF+ group showed delayed early healing compared to the EMD and OFD groups.

**Conclusion:**

The case series (RCT design) suggests a slight advantage for A-PRF+ over EMD and OFD, regarding the regressing of a furcation II to grade I; however this treatment showed delayed early wound healing compared to EMD or OFD.

**Clinical Trial Registration:**

https://www.isrctn.com/, identifier ISRCTN13520922.

## 1. Introduction

It is well known that molar teeth with severe periodontal breakdown run the highest risk of being lost after active periodontal treatment in comparison to other teeth (1, 2). The main reason for their less favorable prognosis is related to the destruction of periodontal attachment and alveolar bone within the furcations of their multiple roots, which creates a poorly accessible ecosystem for new accumulation of pathogenic biofilm after active non-surgical therapy. Also, these furcation defects make proper oral hygiene and professional maintenance difficult. The degree of furcation involvement, as a measure of inter-radicular bone loss, was demonstrated to be a prognostic indicator for the risk of future periodontal attachment loss and even tooth loss (3, 4). A regress of the degree of a furcation involvement of a molar could positively affect the prognosis of the tooth and preserve the masticatory function and the quality of life of the patient.

Several periodontal surgical procedures have been proposed to treat molars with furcation involvement. The most used is the open flap debridement (OFD), in general resulting in recessions and exposed furcation entrances. Nevertheless, molars treated with this technique, demonstrated a good survival rate during an observational period of 5-53 years, even if a high variability in results is present (5). However, an exposed furcation represents a high risk for unexpected complications such as periodontal and/or endodontic complication, root/tooth fracture, root caries and severe sensitivity (6). Furthermore, molars treated with OFD showed a higher rate of re-treatment need, if compared to other surgical strategies (2). In this perspective, the optimal result of a periodontal surgical intervention for periodontally compromised molars would be regenerating the supporting tissues along the roots in the furcation area that have been lost due to the periodontal inflammatory process.

The use of enamel matrix derivative (EMD) in combination with periodontal surgery, demonstrated to increase the chance of achieving (partial) periodontal regeneration compared to OFD and has been proposed as one of the gold standards among the regenerative materials (7, 8). In fact, in periodontal regenerative surgery of furcation defects, randomized clinical trials (RCTs) showed a superiority in furcation grade reduction after the clinical use of EMD compared to guided tissue regeneration (GTR) and OFD with primary closure or coronally advanced flap (9–11). Nevertheless, EMD is a xenogenic material of animal origin (porcine) and this aspect, together with the cost of the product itself, can bring problems in terms of patient acceptance (religious, ethical reasons and/or beliefs). Platelet Rich Fibrin (PRF) has gained a solid position among the available potential materials for regenerative periodontal surgery. Different forms of PRF have been proposed (12). Among these, Advanced-PRF+ (A-PRF+) has demonstrated a beneficial effect in promoting wound healing, due to its slow release of growth factors and cytokines and its dense fibrin network containing active platelets and leucocytes (13–15). These characteristics seem to be especially favorable in the early phase of the wound healing process. Further, another type of PRF preparation, the Leucocyte-PRF (L-PRF), showed better both vertical and horizontal clinical attachment levels (CAL) compared to OFD, even in furcation defects (16–19, 20). The advantages of using PRF preparations in periodontal regeneration procedures is not only the autologous origin and therefore the complete assimilation in patient tissues, but also the immediate and safe availability (13, 21–23).

To date, no studies have been published which compare the use of A-PRF+ with EMD for molar furcation treatment. This is of importance to confirm that A-PRF+ is a suitable alternative to EMD in the surgical treatment of molar teeth with periodontal destruction in the furcation area. Moreover, as A-PRF+ has been shown to possess favorable early wound healing capacities, and this form of autologous regenerative material may be especially suited for the difficult molar furcation surgical treatment; there are no data yet in the literature on this aspect. Therefore, the objective of the current study was to investigate whether the application of A-PRF+ in a regenerative microsurgical approach in molars with furcation involvement results in furcation defect rate reduction, CAL and Bone gain compared with an identical approach employing EMD; the same approach without any material application (designated as the OFD approach) served as control. This study was initially designed as full powered RCT with 3 treatment arms. However, due to the COVID-19 crisis, our clinical trial had to be prematurely terminated, and no re-start was possible. Hence, we present here the 6-month follow-up results for in total 17 consecutive cases that were randomly assigned to the three treatment modalities.

## 2. Materials and methods

### 2.1. Trial design and study progress

**Figure 1:**
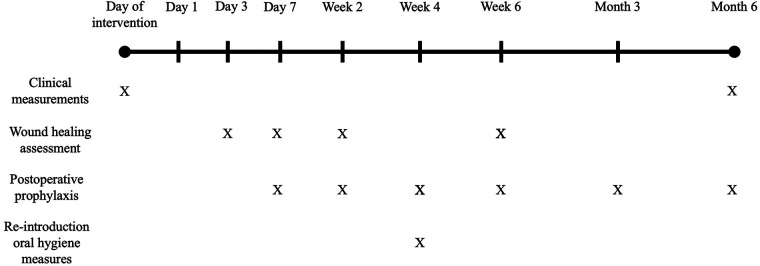
Flowchart and timeline of the study.

The study was designed as a three-arm, double-blind RCT and it was conducted at the Academic Centre for Dentistry Amsterdam (ACTA), Department of Periodontology. The study was approved by the Medical Ethical Committee of the VU Medical Center, Vrije Universiteit Amsterdam (study protocol number: 6265602917). The timeline of the study and the procedures performed at each time point, are provided in Figure 1.

This RCT has been terminated prematurely (March 2020) due to the COVID-19 crisis; the initial lockdown and the subsequent institutional prioritization of capacity of the clinics for acute procedures and teaching purposes, did not allow inclusion of further patients, performance of blood testing and research-related surgical procedures, and their necessary follow-up visits.

The present manuscript has been prepared according to the CONSORT statement for improving the quality of reports of randomized parallel group trials (http://www.consort-statement.org/?o=101).

### 2.2. Participants and inclusion and exclusion criteria

Eligibility screening was conducted on individuals diagnosed with periodontitis who had completed active non-surgical periodontal therapy. The stage of periodontitis was determined based on the 2017 classification (24). Prospective participants who met the inclusion criteria were invited to participate in the study. They were provided with an information sheet detailing the research's content and objectives. A two-week period was provided to consider their participation. Upon agreement, participants were requested to sign an informed consent form.

To be included in the study, patients had to be between 18 and 80 years old, have at least one molar site with a residual furcation involvement grade II (25), and exhibit a residual pocket depth of ≥5 mm after non-surgical therapy. Additionally, their full mouth plaque score (FMPS) had to be less than 20%, and their full mouth bleeding score (FMBS) had to be less than 30%. Several exclusion criteria were implemented during the recruitment process. These included being HIV-positive, having leukopenia or any systemic conditions that hindered wound healing, having allergies to any medications or materials used in the study protocol, being pregnant or lactating, using immunosuppressive medications like corticosteroids or other immune system suppressants on a daily basis, and having taken antibiotics within three months prior to enrolling in the study.

Exclusion criteria specific to the site level were as follows: third molars, lingual furcation sites of mandibular molars, bone loss extending to the apex, endodontically and non-endodontically treated teeth with periapical radiolucency, teeth with vertical fractures or cracks, mobility exceeding one, and furcation involvement grade I or III (25).

### 2.3. Examiner calibration, clinical measurements, and early wound healing assessments

Before the start of the study, a total of 6 non-study periodontitis subjects having at least one grade II furcation defect, were selected from our periodontal clinic. The clinical examiner (NAMR) measured all the clinical parameters described below, in one quadrant of all patients, twice, within 24 h, with at least 45 min between the examinations. The examiner was judged to be reproducible if the percentage of agreement between repeated measurements was at least 90%. The intraclass correlation was calculated for each parameter. For the probing measurements in mm's, there was ≥91% reproducibility allowing a difference of 1 mm; the intraclass correlation for furcation grade was 95%. All periodontal probing was performed using a periodontal probe with a light force of around 0.3 N (William's probe, Hu-Friedy, Chicago, IL, USA). An occlusal hard wax stent was prepared during baseline measurements to determine a reproducible probe position. The stent was kept in the patient file and re-used at the 6-month evaluation.

The following clinical measurements were obtained at the selected furcation site in each patient, at baseline, and at 6 months in the following order (Figure 1): Plaque (Index 0-1-2); Gingival Recession (REC), distance in mm between the cemento-enamel junction and the marginal level of the gingiva; Pocket Depth (PD), distance in mm between the marginal level of the gingiva and the bottom of the pocket; Bleeding (Index 0-1-2); Clinical Attachment Level (CAL), distance in mm between the cemento-enamel junction and the bottom of the pocket; Furcation grade, as I, II, III following the Hamp classification (25); Bone Sounding (Bone-S), distance in mm between the cemento-enamel junction and the bone level in vertical dimension. These parameters were again measured at 6 months postoperatively.

The early wound healing was assessed (DV) using the EHI (26) at day 3, week 1, week 2 and week 6 postoperatively. The index is originally made for interproximal areas, but it is indicated for regenerative procedures. Namely treating interproximal furcations, it can give a good read of the quality of the healing. The examiner was calibrated to establish sufficient intra-examiner reproducibility. For calibration purposes, the EHI was assessed on 4 subjects who did not participate to the study in three separate occasions, clinically the first time and thereafter twice on separate occasions using photographic images of the specific healing sites, at least eight hours apart (27). The kappa value between the second and third measurement on photographs reached 80%.

Intraoral photographs of the area of the periodontal surgery were taken at the time of each assessment (buccal, lingual/palatal, and occlusal site), and photographs were taken additionally of the investigated furcation area. The EHI consists of five categories for the degree of flap closure: (1) Complete flap closure—no fibrin line in the interproximal area; (2) Complete flap closure—fine fibrin line in the interproximal area; (3) Complete flap closure—fibrin clot in the interproximal area; (4) Incomplete flap closure—partial necrosis of the interproximal tissue; (5) Incomplete flap closure—complete necrosis of the interproximal tissue. Examples of the use of this index in molar teeth of patients that participated in the current study are provided in the Supplementary Figure S1.

### 2.4. Blood collection and PRF preparation

In order to maintain patient blinding regarding the type of procedure, a periodontist (LP) collected blood samples from all participants. The blood collection took place through venipuncture of the antecubital fossa immediately before the surgery, specifically for the preparation of A-PRF+ (Advanced Platelet-Rich Fibrin Plus). Each patient provided a total of four tubes of blood, amounting to 40 ml. Two of these tubes, which were sterile plain glass-based vacuum tubes, were used for the A-PRF+ membrane preparation. The other two tubes containing EDTA, which were not intended for A-PRF+ preparation, were sent to a nearby hospital for biochemical analyses of specific blood markers (results not included in this study). The A-PRF+ was prepared using a spinning protocol that involved subjecting the tubes to a relative centrifugation force (RCF max) of 208 g for 8 min using the A-PRF duo Centrifuge from PRF Process (15, 28). Following centrifugation, the A-PRF+ fibrin clot was carefully separated from the red element phase at the base of the tube using sterile pliers. The A-PRF+ clot was then placed in a suitable PRF box (APRF, Nice, France), which exerted constant compression on the clot through the lid (cover of the PRF box). This compression was maintained for 5 min, allowing for the retrieval of A-PRF+ membranes that were uniform in size and thickness. The entire procedure took place in the surgical room, using sterile instruments and a sterile environment, with the patient seated in the dental chair.

### 2.5. Surgical procedure and postoperative instructions and protocol

All surgical procedures were carried out by an experienced periodontist (SB). A microsurgical approach was employed for all surgeries, utilizing a modified or simplified papilla preservation technique based on the width of the interdental space (29–31). If pockets equal or exceeding 5 mm were present at the distal site (retromolar area), a trap-door technique was used, ensuring the preservation of as much tissue as possible in the middle of the ridge. This approach facilitated better coverage and stabilization of the flap. The flap was raised in the gentlest manner possible, according to the papilla preservation technique. Additionally, the flaps were extended apically to the defects and furcation entrances. In cases where necessary, releasing incisions were made on the buccal or lingual side of the most mesial tooth involved in the flap. Thorough degranulation was performed using curettes, including the removal of granulation tissue from furcation-involved areas. Once access to the bone anatomy was achieved, ultrasonic debridement of the roots was carried out with saline cooling, in combination with hand instruments. Up until this point, the surgeon was unaware of the regenerative technique being used, as the treatment allocation was revealed intraoperatively. The person responsible for randomization (LP) opened an envelope containing the treatment allocation, and the surgeon was informed of which of the three approaches (A-PRF+, EMD, or OFD) was to be employed. Care was taken to ensure that the content of the envelope was not disclosed to the patient.

For patients assigned to the OFD group, the surgical area was rinsed with saline (NaCl 0.9%) followed by a one-minute pause, after which the rinsing process was repeated with the same solution. This step was performed to mimic the application of regenerative materials. In the case of A-PRF+ allocation, the two prepared sterile A-PRF+ membranes were trimmed with sterile scissors and compressed as much as possible into the grade II furcation defect of the affected molars. To ensure clot stabilization, a portion of the A-PRF+ membrane was applied to cover the graft material and protect the furcation entrance around the tooth profile. The application of the A-PRF+ membrane material took place within one hour of the start of the surgery. In the case of EMD, the roots were dried, and EMD (Emdogain®, Institut Straumann AG, Basel, Switzerland) was applied and left on the exposed root surface for two minutes. Subsequently, the flap was sutured, and the papilla was repositioned in its original buccal position. Propylene sutures 6–0 (Prolene, Ethicon, Raritan, New Jersey, United States) were used to achieve primary, tension-free closure. The suture technique employed was horizontal mattress with a Laurel loop, with additional single sutures applied if necessary.

Following the surgery, patients were instructed to refrain from any form of brushing in the surgical area. They were advised to rinse with hydrogen peroxide for two minutes followed by a one-minute rinse with chlorhexidine 0.12% (PerioAid 0.12%, Dentaid, Barcelona, Spain) twice a day. Patients were instructed to continue this rinsing regimen for four weeks. The use of analgesics (Paracetamol, also known as acetaminophen, 500 mg, maximum intake of 6 grams per day) was recommended but not prescribed. Sutures were removed after 14 days, and a prophylaxis protocol involving gentle polishing with rubber cups and brushes was performed on day 7, week 2, week 6, month 3 and month 6 after surgery. Patients were instructed to maintain oral hygiene using interdental brushes and an electric toothbrush starting from four weeks postoperatively, at which point chemical plaque control was discontinued.

### 2.6. Statistical analysis

According to previous data in the literature on regenerative procedures in furcation defects (11), we initially considered a clinical difference between the PRF and EMD groups vs. the OFD group in terms of horizontal CAL of 1 mm and a SD of 1 mm. Accepting an α of 0.05 and a power of 80%, the power calculation included 16 participants per group. However, in our clinical setting, using a probe with 3 mm increments (Nabers probe, Hu-Friedy, Chicago, Illinois, US) an accurate 1 mm gain in horizontal CAL appeared not to be feasible, therefore we redefined our primary outcome as the frequency of regress per treatment group from grade II to grade I or less. We first considered that in 30% of the cases when using PRF or EMD we could anticipate a regress of furcation involvement from class II to I, compared to zero % regress in OFD. In that case, 21 patients per group were sufficient (https://clincalc.com/stats/samplesize.aspx). Including a 10% dropout rate, we reached a final number of 23 patients per group to reach sufficient power. All other clinical parameters are secondary outcome measures. Since smokers were not excluded in the study cohort, their distribution was stratified with a specific statistical software (SAS (Statistical Analysis System), SAS institute, North Carolina, USA). Closed numbered envelopes were prepared with the description of the material that was going to be used (A-PRF+, EMD, OFD) and stored in a locked drawer.

The Statistical Package for the Social Sciences (IBM SPSS Statistics Data Editor, Chicago, Illinois, USA) and GraphPad software (GraphPad Prism Version 8.4.1, San Diego, California, USA) were used for data analyses and graphical presentation. For future use of our data, we calculated means and standard deviations for all clinical parameters; these are presented in the supplementary materials. However no statistical testing in the current report was performed.

## 3. Results

### 3.1. Participants and baseline characteristics

**Figure 2:**
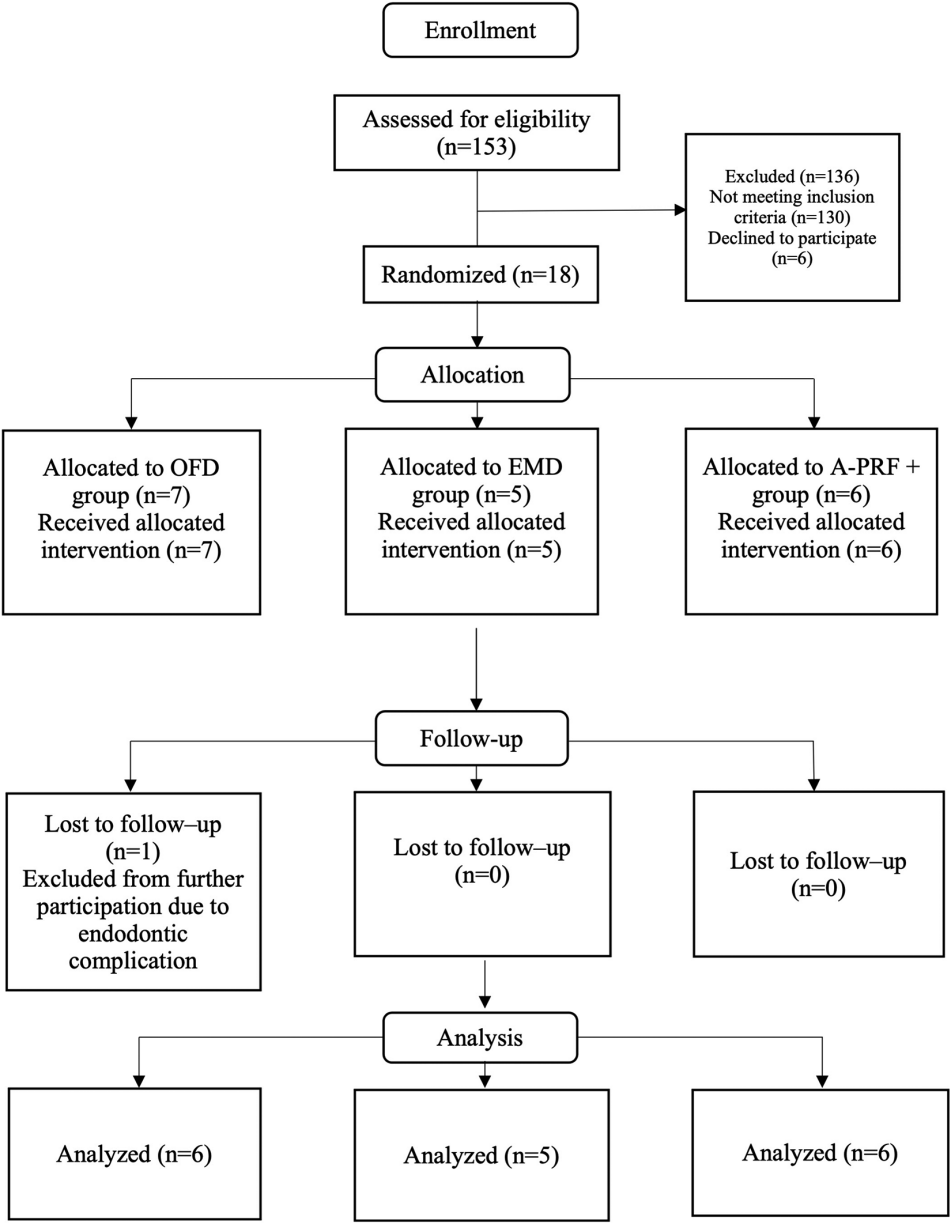
CONSORT flow chart of the study. OFD, open flap debridement; EMD, enamel matrix derivatives; A-PRF+, advanced platelet rich fibrin+.

From October 2018 to March 2020, in total 153 patients were screened for eligibility, of which 18 patients fitted the inclusion criteria and consented to participate in the study (Figure 2). One patient, at 5 weeks post-operative, was excluded from further participation in the study due to an endodontic complication at the tooth that was investigated. Thus, 17 patients were analyzed for this study (Figure 2). Table 1 presents the background and periodontal characteristics of the study population at baseline, 9 females and 8 males participated, with an age range of 33 to 78 years, having from 18 to 32 teeth and from 3 to 12 molar teeth. Fifteen patients were classified as stage III periodontitis, two as stage IV. Also, per patient, the group allocation based on randomization is presented.

**Table 1:** patient and periodontal characteristics at baseline.

Patient^a^	Age	Sex	Smoking (Number of cigarettes per day)	Number Pockets >5 mm	Number of molar teeth^b^	Number of teeth^b^	Periodontitis Stage (24)	Treatment allocation
1	61	F	N	3	8	28	III	A-PRF+
2	42	F	Y (5)	6	8	26	III	EMD
4	33	M	Y (10)	1	8	28	III	OFD
5	71	F	Y (4)	2	8	27	III	A-PRF+
6	78	M	Y (6)	10	4	22	III	A-PRF+
7	57	F	N	4	9	25	III	EMD
8	41	M	Y (10)	3	6	23	III	EMD
9	57	F	N	4	9	25	III	EMD
10	69	F	N	2	3	21	III	OFD
11	63	F	N	15	12	32	III	A-PRF+
12	52	F	N	3	10	27	III	OFD
13	51	M	Y (5)	2	8	28	III	OFD
14	45	M	N	3	4	28	III	OFD
15	70	M	N	1	7	27	III	A-PRF+
16	61	F	N	3	9	18	IV	EMD
17	62	M	N	1	3	18	IV	A-PRF+
18	60	F	N	8	10	26	III	OFD

F, female; M, male; Y, yes; N, no; OFD, open flap debridement; EMD, enamel matrix derivatives; A-PRF+, advanced platelet rich fibrin+.

^a^ Patient 3, although initially assigned to group OFD, was excluded due to acute endodontic complication to the tooth of interest. Refer to Figure 1.

^b^ At inclusion of the study, after non-surgical therapy.

Table 2 presents the tooth characteristics as recorded on the day of the surgery (baseline) for each experimental site per tooth. Based on randomization, in total 13 maxillary (3 in the OFD group, 3 in EMD and 6 in the A-PRF+ group) and 4 mandibular molars (2 in OFD and 2 in EMD group) were included in the study. Due to allocation, five patients had buccal (3 allocated in OFD and 2 in EMD group) furcations and 12 patients had interproximal furcations (3 in OFD, 3 in EMD and 6 in A-PRF+ groups). Regarding the furcation depth at baseline, 3 patients in OFD, 3 patients in EMD and 1 of the patients in A-PRF+ group had furcations with a horizontal attachment loss ≥6 mm (Table 2). Due to the premature termination of the study and consequent randomization according to the protocol, this study has an uneven distribution of type of sites over the 3 types of regeneration procedures.

**Table 2:** Tooth characteristics at baseline (day of the surgery) and furcation at baseline. All teeth had furcation grade II (25).

Group	Patient	Tooth No.	Site	Furcation horizontal depth (mm)^a^
OFD				
	4	47	Buccal	≥6
	10	16	Distal	≥6
	12	16	Mesial	3–5
	13	46	Buccal	3–5
	14	17	Buccal	≥6
	18	26	Distal	3–5
EMD				
	2	26	Mesial	3–5
	7	36	Buccal	≥6
	8	16	Mesial	≥6
	9	16	Mesial	≥6
	16	37	Buccal	3–5
A-PRF+				
	1	16	Mesial	3–5
	5	17	Distal	3–5
	6	16	Distal	3–5
	11	17	Distal	3–5
	15	26	Distal	3–5
	17	27	Mesial	>6

^a^ Furcation grade II defects were categorized as having 3 mm–5 mm or ≥6 mm horizontal depth. Reference was the orifice of the furcation.

Measurements performed with a Nabers probe having 3 mm markings.

### 3.2. Surgical procedures

**Figure 3:**
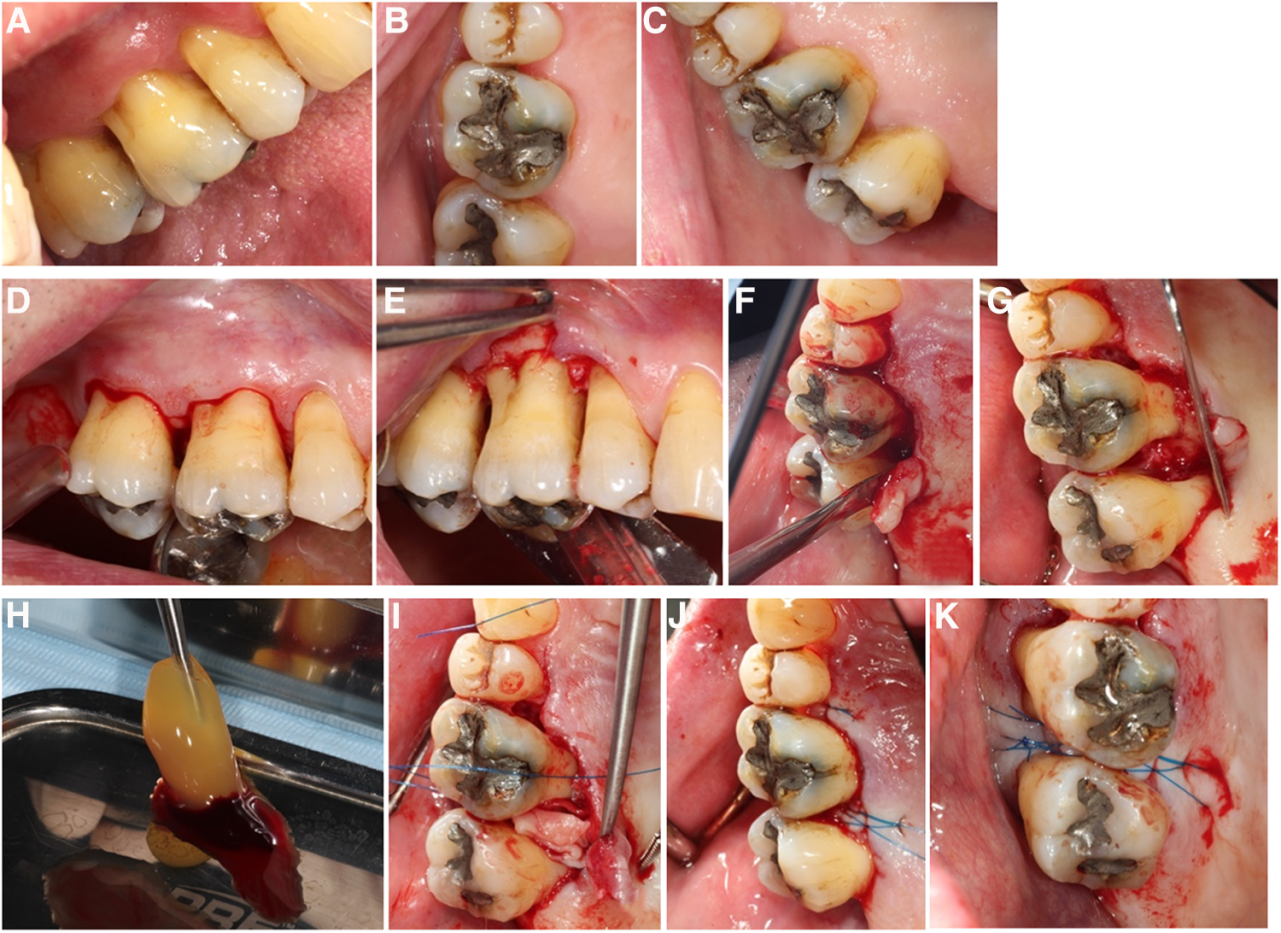
Surgical steps in A-PRF+ regenerative surgery: (**A**) Pre-operative situation, buccal view; (**B**) Pre-operative situation, occlusal view; (**C**) Pre-operative situation, palatal view; (**D**) simplified papilla preservation incision, buccal; (**E**) flap elevation buccal; (**F**) flap elevation lingual, notice the intact interdental papilla; (**G**) surgical site after debridement: mesial furcation of 17 visible; (**H**) A-PRF+ before compression; (**I**) A-PRF+ membrane compressed into the furcation defect; (**J**) internal horizontal mattress suture with Laurel loop, 6–0, lingual view; (**K**) internal horizontal mattress suture with Laurel loop, 6–0, occlusal view.

All surgical procedures were performed according to protocols and no adverse events during the procedures occurred. Blood collections and the handling of the PRF membranes as well as EMD application was uneventful. Subjective healing occurred uneventfully for all participants of the study; objective wound healing results are presented below. Results from intra-surgical measurements are presented in Supplementary Table S2. All molars presented combined bone defects with vertical and horizontal bone loss. The bone loss range was between 3 and 9 mm in vertical direction and between 3 and 7 mm horizontally. The duration of each surgery was recorded, and it ranged from 23 to 72 min (Supplementary Table S2). An example of the surgical procedures performed is depicted in the Figure 3.

### 3.3. Postoperative results of clinical parameters

**Figure 4:**
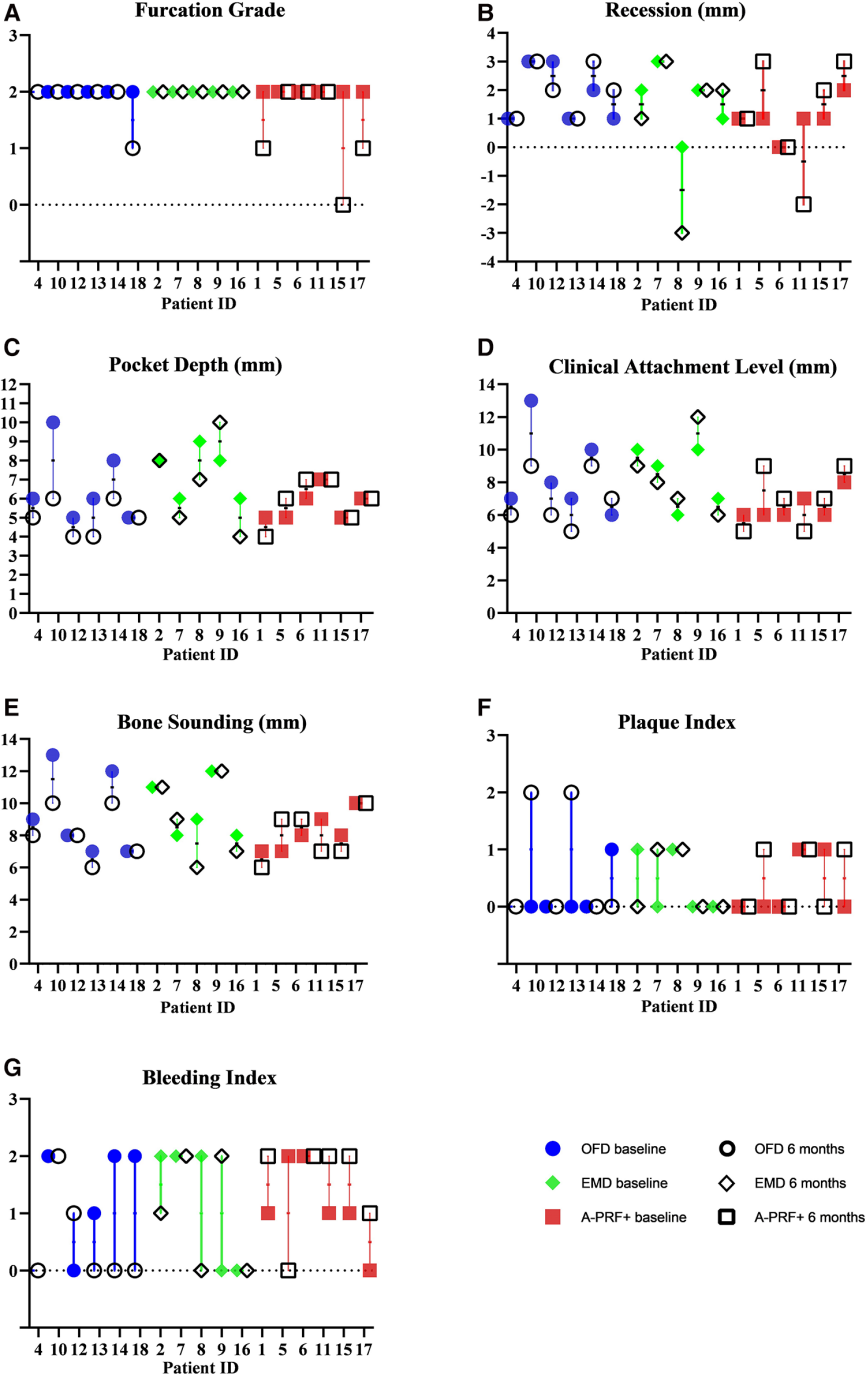
Graphs showing measurements of furcation grade (**A**), recession (**B**), pocket depth (**C**), clinical attachment level (**D**), bone sounding (**E**), plaque index (**F**), bleeding index (**G**) at baseline and 6 months. The values are grouped per treatment modality.

The group with the highest frequency of furcation grade reduction was the A-PRF+ group, where 3 furcations out of 6 improved their grade (2 defects up to grade I and 1 defect moved from grade II involvement to grade 0, the distribution of furcation grade involvement is showed in Table 3). No improvement in furcation degree was visible in the EMD group, while 1 furcation in the OFD group showed a reduction of furcation grade from II to I. Figure 4 show the regression in furcation grade per patient. Table 3 shows the comparison of the measurements of FG at baseline and 6 months.

**Table 3:** Number (percentage) of furcation degree status at 6 months (25).

	Furcation grade 6 months		
	II	I	Closed
OFD	5 (83%)	1 (16%)	
EMD	5 (100%)		
A-PRF+	3 (50%)	2 (32%)	1 (16%)

All molars had a furcation grade II at baseline.

Figure 4 shows the measurements of recession (REC), pocket depths (PD), bone sounding (BoneS), clinical attachments level (CAL), plaque index (PI) and bleeding index (BI) at baseline and 6 months. The values are grouped per treatment modality. 2 patients out 6 in the OFD group presented higher recession values, compared to 1 in the EMD group and 3 in the A-PRF+ group. Some patients show no variation, but 1 patient in the OFD group and 1 in the A-PRF+ group showed a coronal placement of >2 mm of the gum level compared to baseline. 5 furcations out of 6 showed PD reduction in the OFD group, compared to 3 out of 5 in the EMD group and 1 out of 5 in the A-PRF+ group. A gain in CAL was visible in 5 patients out of 6 in the OFD group, 2 out of 5 in the EMD group and 2 out of 6 in the A-PRF+ group. 4 patients out of 6 in the OFD group showed a gain in Bone sounding, compared to 2 out of 5 in EMD group and 3 out of 6 in the A-PRF+ group (Figure 4).

The mean values of the clinical outcomes are presented in Supplementary Table S1.

### 3.4. EHI score

Table 4 demonstrates the EHI of each patient at each follow-up examination until 6 weeks postoperatively. 1 patient (EMD group) did not attend the 1 week follow-up examination because of reported illness. A trend of improvement is visible in all groups, but the EHI score in the A-PRF+ group patient shows a trend of higher values.

**Table 4:** Early wound healing index (EHI) score (26) for each furcation site of each patient at the post-operative period.

Group	Patient	Tooth No.	Site	D3^a^	1W^b^	2W^c^	6W^d^
OFD							
	4	47	Buccal	2	2	1	1
	10	16	Distal	3	2	2	1
	12	16	Mesial	2	2	2	1
	13	46	Buccal	3	2	1	1
	14	17	Buccal	3	2	2	2
	18	26	Distal	3	3	2	1
EMD							
	2	26	Mesial	3	3	2	1
	7	36	Buccal	2	NA	1	2
	8	16	Mesial	3	2	2	2
	9	16	Mesial	3	2	1	1
	16	37	Buccal	2	1	1	1
A-PRF+							
	1	16	Mesial	4	4	5	4
	5	17	Distal	3	3	3	3
	6	16	Distal	2	1	1	1
	11	17	Distal	3	4	4	2
	15	26	Distal	5	5	5	2
	17	17	Mesial	3	2	2	1

Values represent numbers (EHI score).

EHI, Early wound healing index (26); EHI score, 1, complete flap closure—no fibrin line in the interproximal area; 2, complete flap closure—fine fibrin line in the interproximal area; 3, Complete flap closure—fibrin clot in the interproximal area; 4, incomplete flap closure—partial necrosis in the interproximal area; 5, incomplete flap closure—complete necrosis of the interproximal tissue. NA, not applicable.

^a^ 3 days post-operatively.

^b^ 1 week post-operatively.

^c^ 2 weeks post-operatively.

^d^ 6 weeks post-operatively.

**Figure 5:**
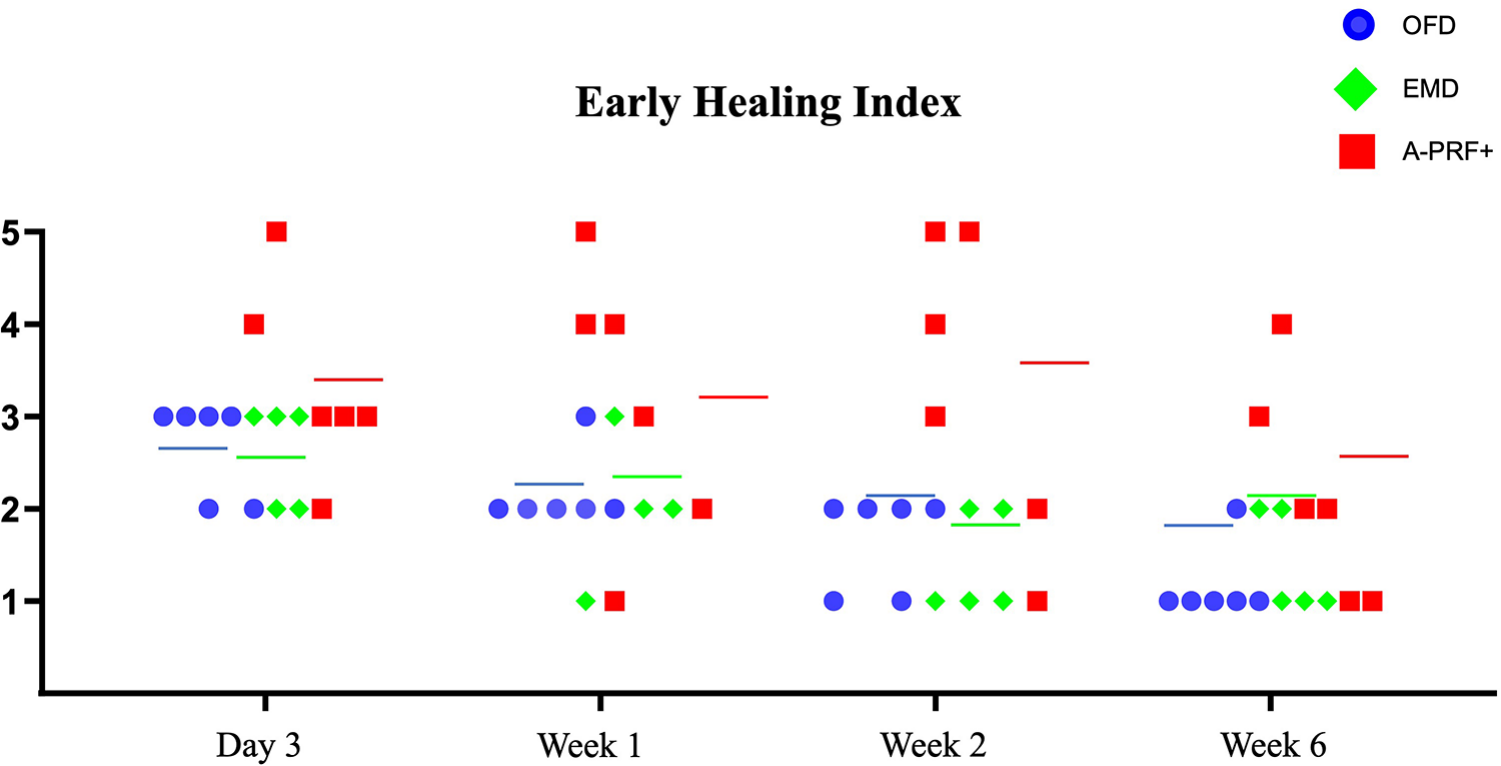
Graph demonstrating the individual data points of early healing Index (26) of the treated furcation sites per group for each follow-up examination (3 days, 1 week, 2 weeks and 6 weeks). Figures on the Y-Axis refer to the EHI categories. The colored line indicates the median per group at every time point. One patient missed the 1-week post-operative examination in the EMD group.

Figure 5 demonstrates the individual data points of Early Healing Index of the treated furcation sites per group for each follow-up examination (3 days, 1 week, 2 weeks and 6 weeks). We can observe that 2 patients form the A-PRF+ group show an incomplete flap closure (EHI score 4 and 5) at the tested furcation sites compared to EMD and OFD groups in each time points. The median EHI shows a trend of delayed wound healing for the A-PRF+ group at each follow-up examination compared to EMD and OFD group. The EMD and OFD showed a similar pattern of healing.

## 4. Discussion

The aim of the current study was to investigate the clinical effects of advanced platelet rich fibrin (A-PRF+) in combination with periodontal regenerative microsurgery in molar furcation defects on furcation grade reduction and clinical parameters (REC, PD, BS, EHI) compared to enamel matrix derivative application (EMD) or open flap debridement (OFD). This is to the best of our knowledge the first time these regenerative materials in furcation defects are compared with each other, even though this study must be considered a pilot. Our study shows that none of the techniques can guarantee a full closure of the furcation defects, but 3 patients out of 6 showed a furcation grade improvement in the A-PRF+ group, compared with 1 patient in the OFD group and none in the EMD group. Clinical values show that 3 patients in the OFD group had an improvement in PD of ≥2 mm, compared with 2 in the EM group and none in the A-PRF+ group. 3 patients in the OFD group show as well ≥2 mm improvement in CAL, while none in the EMD and 1 patient in the A-PRF+ group were present. Concerning the bone sounding, 2 patients showed improvement of ≥2 mm in the OFD group, compared with 1 in the EMD group and 1 in the A-PRF group. The pattern of EHI showed a similar trend regarding the OFD and EMD group, while we observed that at least 2 patients from the A-PRF+ group show an incomplete flap closure (EHI score 4 and 5) at the tested furcation sites compared with EMD and OFD groups up to 2 weeks post operatively.

Previous reports showed that A-PRF+ has biologically favorable qualities. Based on in vitro results, due to its high release of growth factors, PRF can promote neo-angiogenesis, an antibacterial effect, the stimulation of the activity of different cell types (gingival fibroblasts, osteoblasts). Clinically PRF could enhance wound healing and regeneration of angular bony defects (19, 32–36). This study primarily focuses on evaluating the effects of A-PRF+. Based on our extensive pre-clinical experience in recent years, which has highlighted the potential advantages of A-PRF+ in terms of cell proliferation and activity on periodontal fibroblasts and bone cells, we specifically chose A-PRF+ as the test group, excluding other forms of PRF (35, 37, 38). A comprehensive review on the various forms of PRF and for each of them, their common and unique properties, can be found in different reviews (21, 39, 40). EMD can also promote different processes in the healing of periodontal tissues, such as increased protein synthesis, promotion of osteoid matrix formation, neo synthesis of root cement antimicrobial action (41–47).

In our study, 3 patients out of 6 showed regress of furcation involvement in the A-PRF+ group. Previously, different studies investigated the effect of PRF derivatives in furcation areas. A clear advantage compared to control was stated in the studies of Bajaj and Sharma (17, 18). The results from both studies showed improvements in vertical CAL of 2.87 mm ± 0.85 mm and 2.33 mm ± 0.48 mm for the PRF group compared to baseline, which is in contrast compared to our study. Our cohort lost 0.5 mm ± 1.8 mm CAL after the surgical intervention, applying PRF in furcation defects. A possible explanation is that we treated as well interproximal furcations, while the aforementioned studies treated only buccal furcations, more suitable for a better periodontal regeneration (9, 48). Furthermore, PRF showed also higher increase in PD reduction and CAL gain when compared with bone grafts (49, 50). Two recent reviews and meta-analyses showed the advantages of PRF in comparison with OFD and bone graft materials (20, 51). Also here, the results in terms of PD and CAL were in advantage of PRF compared to OFD. The clinical values of our study can be compared with those obtained using EMD in the same clinical situation. Applying EMD, Casarin and colleagues showed improvements in pocket reduction (1.9 mm ± 1.6 mm) and furcation reduction at 24 months compared to OFD (in 50% of cases the furcation involvement regress vs. baseline), while, in our cohort, the EMD group was the one showing the lowest frequency of furcation grade reduction (11). Due to the low number of our cohort, it is possible that known anatomical features (such as the length of the root trunk, the distance of furcation orifice to the bone crest, the root divergence, the infrabony depth of defect in horizontal and vertical direction) were not equally distributed, altering therefore the result. For a better standardization of future studies, it would be optimal to include furcation defects with the same height of bony peaks of the bony defect in relation with the orifice of the furcation. We must observe as well that baseline PD in the A-PRF+ group was somewhat shallower, compared with the other groups. This might result in a lower pocket reduction, but as well in a better healing result for the horizontal component of the defect. The study lacks radiographic analyses in the diagnosis and follow-up of participants, which could provide more precise assessment of periodontal defects and treatment outcomes. However, traditional 2-dimensional radiographs have limited effectiveness in detecting interproximal furcation reduction due to the masking effect of the cortical bone plate on infrabony defects. While incorporating cone-beam computed tomography (CBCT) to measure bone differences between different time points could be considered an ideal approach, the potential advantages of this technique in the scientific literature remain controversial (52, 53). Although we did collect radiographs at baseline and re-evaluation, we acknowledge the limitations of our study design, which led us to primarily focus on clinical and wound healing parameters.

The type of surgical technique played certainly a role in the results of REC, PD and CAL. In fact, we noticed a trend of negative recessions, deeper pockets, and attachment loss in the A-PRF+ group at 6 months compared to baseline and compared to the other groups. Furthermore, the EHI reached a trend of higher scores in the early time points in the A-PRF+ group. This can be explained by the surgical technique chosen for the study. We performed a papilla preservation technique, aimed to closing as much as possible the interdental tissues. Materials such as EMD, having no volume and being fluidic, are better suited for this. But using A-PRF+, and applying the compressed fibrin membranes, may result in a more coronal repositioning of the flap and sometimes even in a slight opening of the wound in the first week. This seems to originate from the hydrophilic behavior of PRF and A-PRF+ membranes, which probably increase in volume after their application, after pulling the flap and the tissues coronally. The slight opening of the papilla immediately after surgery is as well responsible of the worse EHI in the A-PRF+ group, showing from the beginning higher values compared to the other groups. Possibly, a papilla preservation approach is not indicated if combined with PRF. For 3 out of 6 included sites, A-PRF+ resulted in a reduction of the furcation grade. This is explainable with the extra space provided by the coronal displacement of tissues during flap management, that allowed in any case a healing of deepest part of the defect. Nevertheless, an ideal regenerative material should demonstrate both space-maintaining ability and biological properties that promote the migration of autologous cells capable of regenerating lost tissues. However, neither EMD nor A-PRF+ is mechanically sufficient to achieve this goal.

Two other clinical aspects need attention. Smokers (*n* = 6) were included as well in the study. It is important to acknowledge that smoking can have a substantial impact on periodontal treatment outcomes as well as the utilization of platelet concentrates. The negative effect of smoking is widely known, in particular after periodontal regeneration techniques (54). Although a direct correlation between smoking and the quality of platelet concentrates is yet to be established, evidence suggests that smoking has the potential to influence mechanical and biological effect of PRF materials (55). However, there is currently a lack of extensive literature on this topic. To balance the relative effect of smoking, we stratified the patients during randomization, enrolling 2 smokers per group. Further, the distribution of upper and lower molars is as well a point of interest. There is a clear skewed distribution between mandibular furcations in the groups, where the A-PRF+ group has no mandibular furcations. This may have had an impact in the early wound healing as well, where interproximal sites are more prone to an opening of the sutured flap.

The positive aspects of our study are the randomization, the stratification and the blinding of both participants and examiners. Measurements were standardized using coronal wax stents, to minimize differences in the position of the periodontal probe. Patients underwent a strict post-operative protocol, in which post-operative instruction, plaque control and inspection of the quality of healing were performed at day 3, 7, 14, and at month 3 and 6. The plaque control of the operated area was on good standards for every patient.

Among the limitations, the number of patients is the most important one, and influenced the distribution of surgical sites and the final generalizability of the study. Unfortunately, the study was prematurely terminated in March 2020 due to the COVID-19 pandemic. Only post-operative follow-up of included patients was possible while the clinics of ACTA stopped clinical research.

To conclude, treatment modalities to reduce horizontal depth of furcation defects can improve the long-term prognosis of the involved molars, but furcation closure procedures are yet still unpredictable. Our study is not an exception. We observed improvements in some patients, but these results were not consistent in all the study participants.

## 5. Conclusions

From the current case series with an RCT design we cannot conclude that the application of a PRF preparation (A-PRF+) associated with micro-surgical technique is superior to OFD or EMD comparing parameters in vertical dimension. Nevertheless, in our study furcation grade regression from II to I or closure occurred in 3 out of 6 patients in the A-PRF+ group, while the regress was 1 and 0 in the OFD and EMD group respectively. This may suggest a slight advantage for A-PRF+ over EMD and OFD, regarding the regressing of a furcation II to grade I or 0. Concerning the healing, A-PRF+ showed a different pattern compared to OFD and EMD, having a delayed healing at all short-term postoperative time points. It might be considered that for the application PRF membranes a different surgical technique must be used.

Because we present here a cases series, the results cannot be generalized. Further RCTs are needed to draw a definitive conclusion regarding the effects of A-PRF+ when applied in periodontal regeneration for molar furcation defects.

## Data Availability

The raw data supporting the conclusions of this article will be made available by the authors, without undue reservation.
