# Supplementary figures and images for: Effects of Advanced Platelet Rich Fibrin (A-PRF+), Enamel Matrix Derivative (EMD) and Open Flap Debridement on clinical and wound healing parameters in molar furcation sites: A case series from a RCT study
Source: Front Dent Med. 2023 Jul 31;4:1223217. doi: 10.3389/fdmed.2023.1223217 (PMC11811778; doi:10.3389/fdmed.2023.1223217)

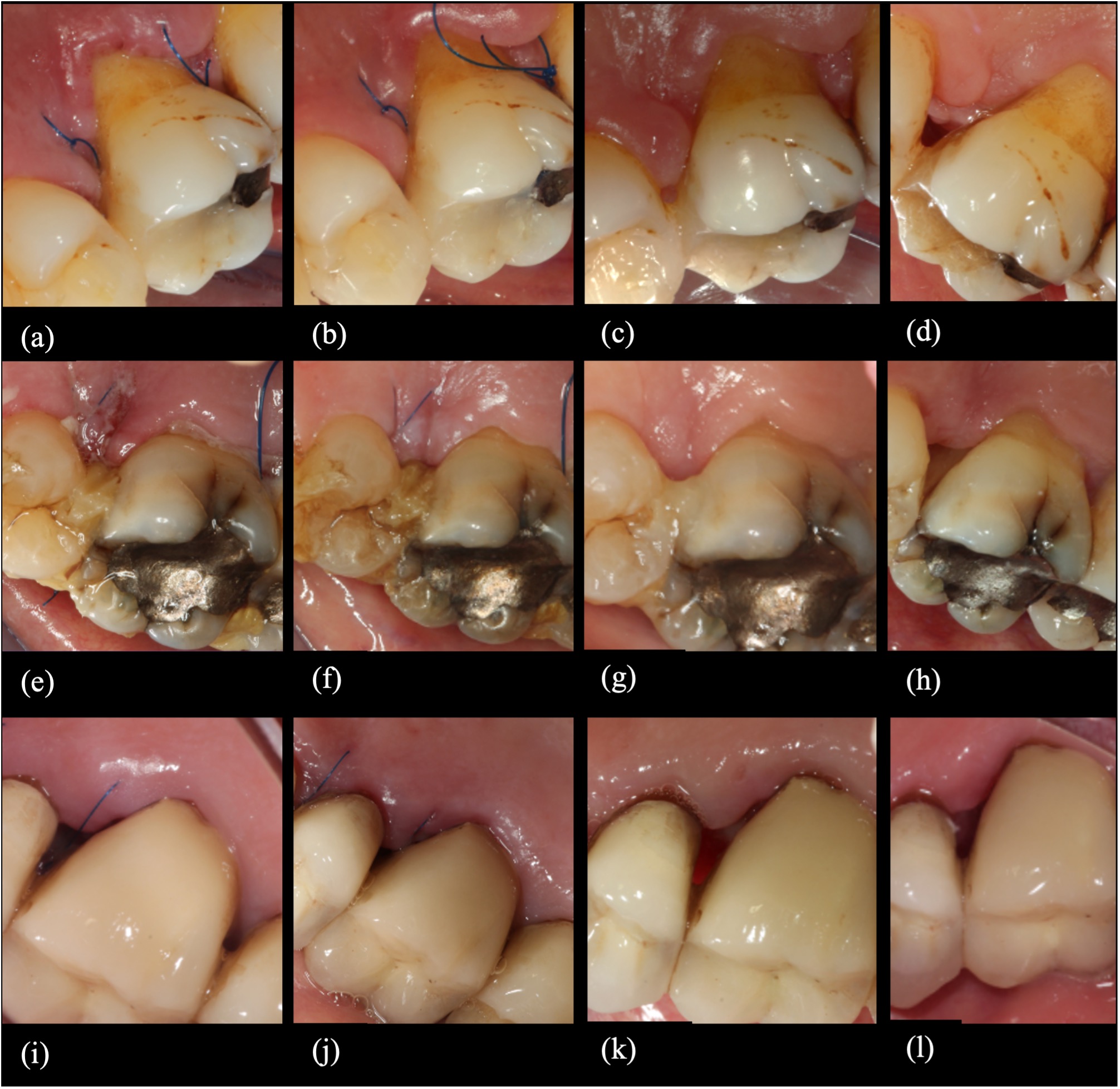

Supplement: Supplementary file 1 [file Image1.jpeg]
