# Supplementary material for: Effects of Advanced Platelet Rich Fibrin (A-PRF+), Enamel Matrix Derivative (EMD) and Open Flap Debridement on clinical and wound healing parameters in molar furcation sites: A case series from a RCT study
Source: Front Dent Med. 2023 Jul 31;4:1223217. doi: 10.3389/fdmed.2023.1223217 (PMC11811778; doi:10.3389/fdmed.2023.1223217)
Supplement: Supplementary file 2 [file Datasheet1.docx]

**Supplementary Material**

|  |  | Baseline | 6 months  follow-up | 0-6 months difference |
| --- | --- | --- | --- | --- |
| REC^a^ |  |  |  |  |
|  | OFD | 1.8 (1.0) | 2.0 (0.9) | -0.2 (0.7) |
|  | EMD | 1.6 (1.4) | 1.0 (2.3) | 0.6 (1.5) |
|  | A-PRF+ | 1.0 (0.6) | 1.2 (1.9) | -0.2 (1.7) |
| PD^b^ |  |  |  |  |
|  | OFD | 6.7 (1.9) | 5.0 (0.9) | 1.7 (1.4) |
|  | EMD | 7.4 (1.3) | 6.8 (2.4) | 0.6 (1.7) |
|  | A-PRF+ | 5.7 (0.8) | 5.8 (1.2) | -0.2 (0.7) |
| CAL^c^ |  |  |  |  |
|  | OFD | 8.5 (2.4) | 7.0 (1.7) | 1.5 (1.6) |
|  | EMD | 8.4 (1.6) | 8.4 (2.3) | 0.0 (1.4) |
|  | A-PRF+ | 6.5 (0.8) | 7.0 (1.8) | -0.5 (1.8) |
| BoneS^d^ |  |  |  |  |
|  | OFD | 9.3 (2.6) | 8.2 (1.6) | 1.7 (1.7) |
|  | EMD | 9.0 (1.6) | 8.8 (2.6) | 1.0 (1.2) |
|  | A-PRF+ | 8.2 (1.2) | 8.0 (1.5) | 0.2 (1.5) |
| BI ≥1 at the surgical site |  |  |  |  |
|  | OFD | 1. % (4) | 33 % (2) | -33% |
|  | EMD | 60 % (3) | 60 % (3) | 0 % |
|  | A-PRF+ | 83 % (5) | 83 % (5) | 0 % |
| PI ≥1 at the surgical site |  |  |  |  |
|  | OFD | 16 % (1) | 33 % (2) | -17 % |
|  | EMD | 40 % (2) | 40 % (2) | 0 % |
|  | A-PRF+ | 1. % (2) | 50 % (3) | -17 % |

**TABLE S1** Means (± SD) of recessions (REC), probing depth (PD), and vertical clinical attachment level (CAL) and vertical bone sounding (BoneS) at the surgical site at baseline and follow-up; percentage of positive bleeding index (BI) and plaque index (PI) at the surgical site at baseline and follow-up.

^a^REC (Recession): distance in mm between the cementoenamel junction and the gingival margin.

^b^PD (Pocket Depth): distance in mm between the gingival margin and the attachment of the periodontal ligament.

^c^CAL (Clinical Attachment Level): distance in mm between cementoenamel junction and the attachment of the periodontal ligament.

^d^BoneS (Bone Sounding): distance in mm between the cementoenamel junction and the bone level in vertical dimension.

**TABLE S2** Intra-surgical measurements and time of intervention.

| Group | Tooth No. | HBL^a^ | VBL^b^ | BD^c^ | FBL^d^ | Duration of intervention^e^ |
| --- | --- | --- | --- | --- | --- | --- |
| OFD |  |  |  |  |  |  |
| 4 | 47 | 6 | 6 | 2 | 4 | 43 |
| 10 | 16 | 7 | 9 | 5 | 3 | 56 |
| 12 | 16 | 5 | 7 | 0 | 4 | 40 |
| 13 | 46 | 5 | 6 | 2 | 3 | 32 |
| 14 | 17 | 7 | 8 | 3 | 1 | 51 |
| 18 | 26 | 5 | 6 | 3 | 2 | 28 |
| EMD |  |  |  |  |  |  |
| 2 | 26 | 4 | 5 | 2 | 2 | 64 |
| 7 | 36 | 6 | 7 | 1 | 5 | 46 |
| 8 | 16 | 6 | 9 | 5 | 0 | 41 |
| 9 | 16 | 7 | 3 | 7 | 2 | 72 |
| 16 | 37 | 5 | 5 | 3 | 0 | 25 |
| A-PRF+ |  |  |  |  |  |  |
| 1 | 16 | 3 | 3 | 1 | 2 | 62 |
| 5 | 17 | 3 | 7 | 2 | 4 | 39 |
| 6 | 16 | 3 | 5 | 2 | 2 | 67 |
| 11 | 17 | 5 | 7 | 4 | 0 | 52 |
| 15 | 26 | 5 | 6 | 2 | 3 | 37 |
| 17 | 27 | 6 | 9 | 3 | 3 | 54 |

^a^HBL (horizontal bone level): distance in mm between the projection of the entrance of the furcation and the bone level in horizontal dimension.

^b^VBL (vertical bone level): distance in mm between the cemento-enamel junction and the bone level in vertical dimension.

^c^BD (bone defect): depth in mm of the bone defect in vertical dimension.

^d^FBL (furcation bone level): distance in mm between the orifice of the furcation and the bone level in vertical dimension.

^e^Duration of intervention: total duration in minutes of surgical procedure (intervention)

**FIGURE S1** Examples of early wound healing of one patient per group as assessed at the follow-up examinations. For all these 3 patients the 16 mesial site was assessed.

a) APR12 (OFD) at 3 days, EHI 2; b) APR12 at 1 week, EHI 2; c) APR12 at 2 weeks, EHI 2; d) APR12 at 6 weeks, EHI 1.

e) APR09 (EMD) at 3 days, EHI 3; f) APR09 at 1 week, EHI 2; g) APR09 at 2 weeks, EHI 1; h) APR09 at 2 weeks, EHI 1.

i) APR01 (A-PRF) at 3 days, EHI 4; j) APR01 at 1 week, EHI 4; k) APR01 at 2 weeks, EHI 5; l) APR01 at 6 weeks, EHI 4.
